# Giant Morgagni hernia with multivisceral involvement: a case report of successful surgical repair in an elderly patient

**DOI:** 10.1093/jscr/rjag242

**Published:** 2026-04-09

**Authors:** Tarek Bulbul, Philippe Attieh, Mustapha Allouche

**Affiliations:** General Surgery Department, University of Balamand, Beirut 1300, Lebanon; General Surgery Department, University of Balamand, Beirut 1300, Lebanon; General Surgery Department, University of Balamand, Beirut 1300, Lebanon; Chairman of Surgery Department, NINI Hospital, Tripoli 1300, Lebanon

**Keywords:** Morgagni hernia, diaphragmatic hernia, abdominal pain, elderly patient, laparoscopic surgery

## Abstract

Morgagni hernia (MH) is a rare congenital diaphragmatic defect, representing 2%–4% of adult diaphragmatic hernias. It occurs through the sternocostal triangle, usually on the right side, and may remain undiagnosed because of nonspecific or absent symptoms. We report, a 75-year-old woman with hypertension and dyslipidemia presented with 3 days of progressive upper abdominal pain, dysphagia, nausea, vomiting, and poor oral intake. Examination revealed decreased breath sounds and bowel sounds over the right lung base. Chest radiography demonstrated right diaphragmatic elevation with a colonic air–fluid level. Computed tomography confirmed a large right-sided MH containing the gastric antrum, pylorus, and colon without obstruction. Surgical repair with mesh was performed, and recovery was uneventful. In conclusion, early imaging and surgical management are essential to prevent complications.

## Introduction

Morgagni hernia (MH) is a rare congenital diaphragmatic hernia representing 2%–4% of adult cases [1]. It occurs through an anterior retrosternal defect in the sternocostal triangle, most commonly on the right side due to the protective effect of the pericardium on the left hemidiaphragm; bilateral cases are extremely uncommon [1]. Predisposing factors include pregnancy, trauma, obesity, chronic constipation, and chronic cough [1]. Although some MHs are detected incidentally, many present with nonspecific respiratory or gastrointestinal symptoms such as epigastric or retrosternal pain [1]. The omentum and colon are most frequently involved, and bowel herniation increases the risk of obstruction, strangulation, and ischemia. Surgical repair remains the standard treatment in adults [1].

## Case presentation

A 75-year-old female with hypertension and dyslipidemia, no prior surgeries, no smoking history, and no known allergies presented with progressively worsening epigastric pain and dysphagia of 3 days’ duration. She reported nausea, two episodes of non-bloody, non-bilious vomiting, postprandial discomfort, and reduced oral intake.

On admission, heart rate was 114 bpm, blood pressure 105/75 mmHg, temperature 37°C, and oxygen saturation 94% on room air. Examination revealed decreased air entry at the right lung base with audible bowel sounds. Laboratory tests including complete blood count, electrolytes, renal and liver function tests, and serum lactate were normal, with no leukocytosis, electrolyte imbalance, hepatic dysfunction, or metabolic acidosis. Normal lactate levels and absence of clinical signs of vascular compromise reduced the likelihood of bowel ischemia at presentation.

**Figure 1:**
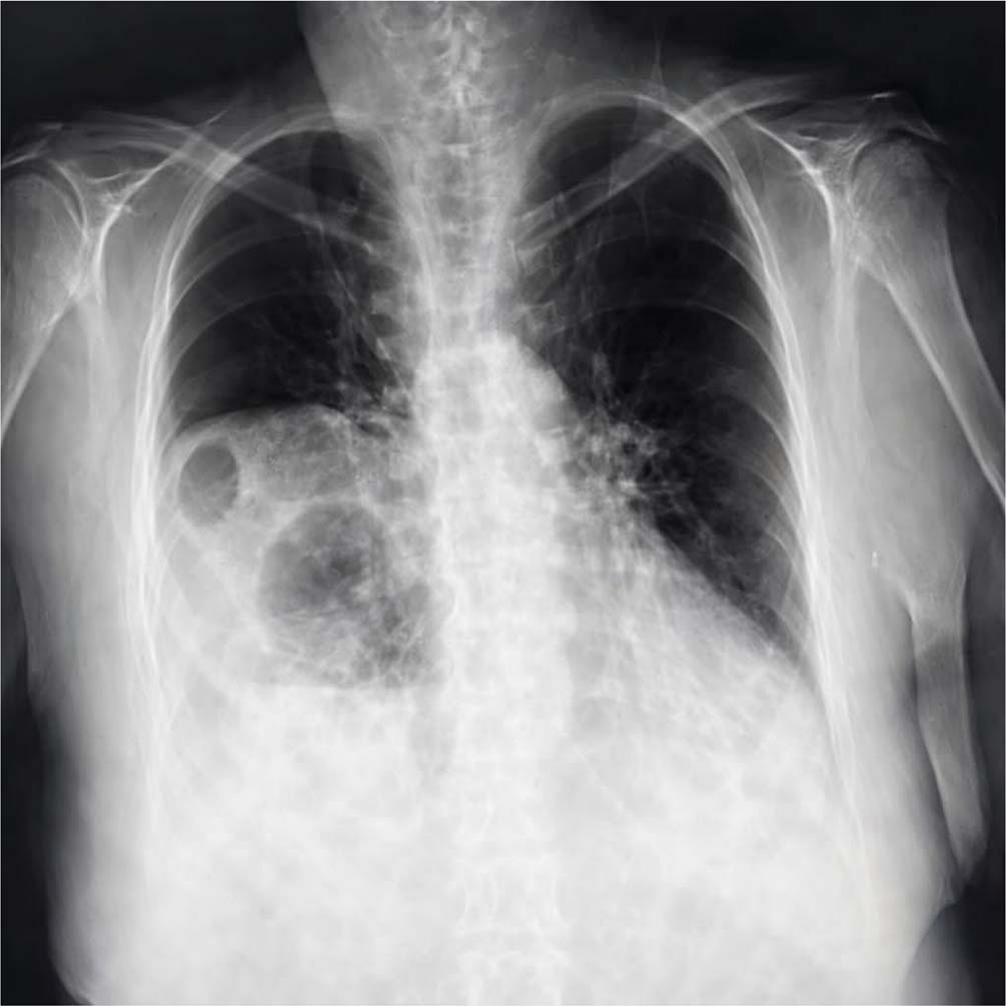
Chest X-ray showing elevation of the right diaphragmatic cupola, eventration (blue arrow), obliteration of the left subdiaphragmatic recess and sub diaphragmatic colonic air fluid level.

**Figure 2:**
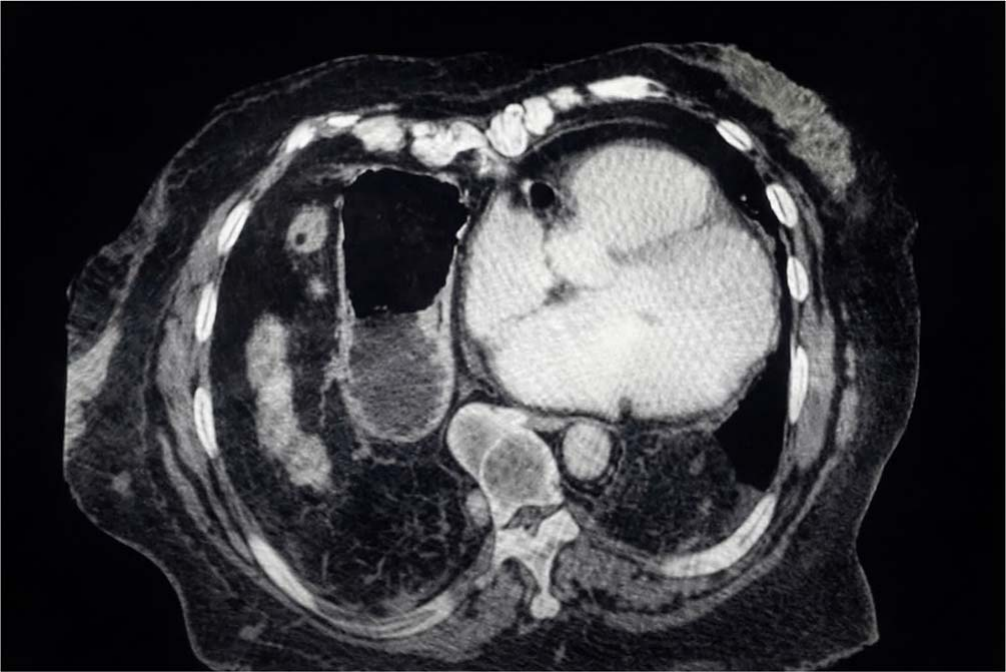
CT scan showing a large right anterolateral diaphragmatic hernia with a maximal *craniocaudal extent*.

Chest X-ray (Fig. 1) showed elevation of the right hemidiaphragm, diaphragmatic eventration, obliteration of the left subdiaphragmatic recess, and a subdiaphragmatic colonic air-fluid level. Computed tomography (CT) (Fig. 2) confirmed a large right anterolateral diaphragmatic hernia with a craniocaudal extent of ~16 cm and a hernia neck measuring 2 × 5 cm. Measurements were obtained from multiplanar CT reconstructions and reflected the hernia sac size and defect width. A small left-sided fat-containing diaphragmatic hernia (collar 12 mm) and a right uncomplicated inguinal hernia containing distal ileum were also identified.

The patient underwent laparoscopic repair under general anesthesia (Video 1). Herniated gastric and colonic contents were reduced into the abdominal cavity. The right MH sac was dissected and excised to minimize recurrence and seroma formation. The diaphragmatic defect (2 × 5 cm) was closed primarily using non-absorbable interrupted sutures and reinforced with a 10 × 15 cm composite synthetic mesh placed in an intraperitoneal onlay position (IPOM), ensuring adequate overlap. The small contralateral fat-containing hernia was repaired during the same procedure. The asymptomatic right inguinal hernia was not addressed to limit operative time and morbidity in this elderly patient.

Total operative time was 90 min, with estimated blood loss of 150 mL. No intraoperative complications occurred. Postoperatively, the patient passed flatus on day one, tolerated mild analgesics, and advanced diet progressively. She was discharged on postoperative day three with analgesics, antiemetics, and a proton pump inhibitor, and scheduled for outpatient follow-up in 10 days.

## Discussion

Diaphragmatic hernias occur when abdominal contents protrude into the thoracic cavity through a diaphragmatic defect. MH arises through the anterior retrosternal sternocostal triangle between the xiphoid process and costal diaphragm. Right-sided defects are termed MH, while left-sided defects are referred to as Larrey hernias [1].

In adults, MH accounts for 2%–4% of congenital diaphragmatic hernias and is more common on the right due to left-sided pericardial protection [1]. Bilateral herniation is rare [1]. The condition results from developmental defects in diaphragm formation, with increased intra-abdominal pressure from pregnancy, trauma, obesity, chronic constipation, or chronic cough acting as precipitating factors.

Presentation in adults is often nonspecific. Respiratory symptoms may include dyspnea or recurrent infections, while gastrointestinal manifestations include epigastric pain, retrosternal discomfort, nausea, vomiting, or dysphagia [1]. The omentum and colon are most commonly involved. Isolated omental herniation is often asymptomatic, whereas involvement of colon, small bowel, or stomach increases the risk of obstruction, strangulation, ischemia, and perforation. For this reason, surgical repair is recommended in adults regardless of symptom severity [1].

A review of reported adult MH cases [2–12] (Table 1) shows predominance in elderly patients and presentation with nonspecific gastrointestinal or respiratory complaints. CT is the diagnostic modality of choice, providing accurate delineation of hernia contents and defect size. Colon and omentum are most frequently identified, with gastric involvement less common. Minimally invasive approaches have increasingly become preferred in stable patients, demonstrating favorable outcomes.

**Table 1:** Summary of reported adult MH cases in the literature.

**Author (year)**	**Age / sex**	**Presenting symptoms**	**Imaging modality**	**Hernia contents**	**Surgical approach**	**Outcome**
Ibba *et al*. (2024) [2]	72 / Female	Dyspnea after COVID-19 pneumonia	CXR, CT chest	Transverse colon, omentum	Thoracotomy	Uneventful recovery
Choy *et al*. (2019) [3]	81 / Male	Dyspnea, constipation	CT chest/abdomen	Colon	Laparoscopic repair	Recovered
Assi *et al*. (2024) [4]	Elderly / Female	Hypoxemic respiratory distress	CT chest	Omentum, colon	Laparoscopic repair	Clinical improvement
Leite *et al*. (2020) [5]	Elderly / Female	Dyspnea	CT chest	Colon, omentum	Laparoscopic repair	Uneventful recovery
Khan *et al*. (2017) [6]	65 / Male	Chronic constipation	CXR, barium study	Colon	Laparotomy	Good outcome
Zhang *et al*. (2021) [7]	74 / Male	Dysphagia, epigastric pain	CT with 3D reconstruction	Colon, omentum	Laparoscopic repair	Recovered
Kim *et al*. (2017) [8]	84 / Female	Chronic dyspepsia	CXR, CT abdomen	Omentum	Conservative → surgery	Symptom resolution
Rajkumar *et al*. (2022) [9]	84 / Female	Hematemesis, abdominal distension, dyspnea	CT abdomen/pelvis	Colon	Laparoscopic mesh repair	Uneventful recovery
Aslam *et al*. (2021) [10]	64 / Female	Nausea, vomiting, regurgitation	CT chest	Stomach, colon	Laparotomy	Recovered
O’Brien *et al*. (2017) [11]	83 / Female	Abdominal pain, coffee-ground emesis	CT chest/abdomen	Stomach, small bowel	Bilateral mesh repair	Favorable outcome
Nguyen *et al*. (2023) [12]	81 / Female	Vomiting, bowel obstruction	CT chest/abdomen	Cecum, ascending colon	Laparotomy	Recovered
Present case	75 / Female	Epigastric pain, dysphagia, vomiting	CXR, CT	Stomach, colon	Laparoscopic repair with mesh	Uneventful recovery; no recurrence

This case is notable for the coexistence of a large right-sided MH containing both gastric and colonic segments, a smaller contralateral fat-containing diaphragmatic hernia, and a right inguinal hernia in an elderly female presenting primarily with gastrointestinal symptoms. Bilateral involvement is exceptionally uncommon, and the additional inguinal hernia further increased anatomical complexity.

The laparoscopic approach was selected based on hemodynamic stability, absence of ischemia, and the advantages of minimally invasive surgery in elderly patients, including reduced postoperative pain, shorter hospitalization, and faster recovery. Mesh reinforcement was chosen rather than primary closure alone due to defect size, chronicity, and the need to minimize tension and recurrence risk, consistent with current adult MH repair recommendations.

The inguinal hernia was not repaired simultaneously, as it was asymptomatic and uncomplicated. A staged strategy was favored to reduce operative time and perioperative risk. The mildly reduced oxygen saturation on admission was likely attributable to thoracic compression and ventilation–perfusion mismatch from intrathoracic displacement of abdominal viscera, resolving after surgical reduction.

Postoperative follow-up consisted of clinical evaluation at 10 days, with planned chest imaging at 6–12 months to assess for recurrence, although standardized surveillance protocols for adult MH repair are lacking.

This report has limitations inherent to single-case studies. Findings cannot be generalized to broader populations, and follow-up was limited to the early postoperative period, precluding long-term recurrence assessment. Furthermore, no comparative analysis between laparoscopic and open techniques can be derived. Larger case series and comparative studies are required to clarify optimal surgical approaches and long-term outcomes in adult MH repair.

## Conclusion

MH is a rare diaphragmatic defect in adults that may present predominantly with gastrointestinal symptoms such as dysphagia, epigastric pain, and vomiting. CT imaging is essential for diagnosis and operative planning. Laparoscopic repair with mesh reinforcement can be safely performed in selected stable patients, even in the absence of obstruction or ischemia. Given the rarity of bilateral involvement and coexistence with additional hernias, comprehensive imaging and individualized surgical planning are critical. Larger studies are needed to better define optimal management strategies and long-term recurrence outcomes.

## Supplementary Material

Supplementary_Video_1_rjag242

VID-20250928-WA0024_rjag242
