# Supplementary material for: Giant Morgagni hernia with multivisceral involvement: a case report of successful surgical repair in an elderly patient
Source: J Surg Case Rep. 2026 Apr 9;2026(4):rjag242. doi: 10.1093/jscr/rjag242 (PMC13070391; doi:10.1093/jscr/rjag242)
Supplement: Supplementary_Video_1_rjag242 [file supplementary_video_1_rjag242.docx]

**Video 1.** Laparoscopic surgical repair with excision of the right MH sac and mesh placement along with left diaphragmatic left hernia repair
